# Exploring Curriculum Considerations to Prepare Future Radiographers for an AI-Assisted Health Care Environment: Protocol for Scoping Review

**DOI:** 10.2196/60431

**Published:** 2025-03-06

**Authors:** Chamandra Kammies, Elize Archer, Penelope Engel-Hills, Mariette Volschenk

**Affiliations:** 1 Department of Health Professions Education Faculty of Medicine and Health Sciences Stellenbosch University Cape Town South Africa; 2 Department of Medical Imaging and Radiation Sciences Faculty of Health Sciences University of Johannesburg Johannesburg South Africa; 3 Professional Education Research Institute Cape Peninsula University of Technology Cape Town South Africa

**Keywords:** artificial intelligence, machine learning, radiography, education, scoping review

## Abstract

**Background:**

The use of artificial intelligence (AI) technologies in radiography practice is increasing. As this advanced technology becomes more embedded in radiography systems and clinical practice, the role of radiographers will evolve. In the context of these anticipated changes, it may be reasonable to expect modifications to the competencies and educational requirements of current and future practitioners to ensure successful AI adoption.

**Objective:**

The aim of this scoping review is to explore and synthesize the literature on the adjustments needed in the radiography curriculum to prepare radiography students for the demands of AI-assisted health care environments.

**Methods:**

Using the Joanna Briggs Institute methodology, an initial search was run in Scopus to determine whether the search strategy that was developed with a library specialist would capture the relevant literature by screening the title and abstract of the first 50 articles. Additional search terms identified in the articles were added to the search strategy. Next, EBSCOhost, PubMed, and Web of Science databases were searched. In total, 2 reviewers will independently review the title, abstract, and full-text articles according to the predefined inclusion and exclusion criteria, with conflicts resolved by a third reviewer.

**Results:**

The search results will be reported using the PRISMA-ScR (Preferred Reporting Items for Systematic Reviews and Meta-Analyses Extension for Scoping Reviews) checklist. The final scoping review will present the data analysis as findings in tabular form and through narrative descriptions. The final database searches were completed in October 2024 and yielded 2224 records. Title and abstract screening of 1930 articles is underway after removing 294 duplicates. The scoping review is expected to be finalized by the end of March 2025.

**Conclusions:**

A scoping review aims to systematically map the evidence on the adjustments needed in the radiography curriculum to prepare radiography students for the integration of AI technologies in the health care environment. It is relevant to map the evidence because increased integration of AI-based technologies in clinical practice has been noted and changes in practice must be underpinned by appropriate education and training. The findings in this study will provide a better understanding of how the radiography curriculum should adapt to meet the educational needs of current and future radiographers to ensure competent and safe practice in response to AI technologies.

**Trial Registration:**

Open Science Framework 3nx2a; https://osf.io/3nx2a

**International Registered Report Identifier (IRRID):**

PRR1-10.2196/60431

## Introduction

Radiography is arguably one of the most technologically advanced health care disciplines, encompassing various technologies that continuously evolve with advancements in computing power and human knowledge [1,2]. Although the use of technology has always been extensive in the profession, recent technological advancements have focused on incorporating complex machine learning algorithms, which have led to a change in clinical protocols that affect patient outcomes and radiography practice [3-5]. Artificial intelligence (AI) technologies play an increasingly important role in clinical radiography with applications such as scheduling patients, image interpretation and reporting, vetting of examinations, patient positioning, image generation and reconstruction, radiation therapy dosimetry, and image postprocessing [4,6-8]. As the number of AI applications increases in clinical practice [9], it is postulated that the practitioner role may change and expand, and these new roles will require modifications to the competencies and educational requirements of current and future practitioners [4].

The increasing integration of AI technologies in radiography practice raises significant questions about the influences of AI technology on radiography practice and the subsequent ways in which radiography education may be required to change. Recent reviews have explored AI’s current and potential applications in radiography practice [5] and examined the AI educational programs available to radiography staff globally [1]. Furthermore, research undertaken in the United Kingdom, Africa, and the Middle East exploring AI topics linked to education in radiography has focused on equipping practitioners for successful AI adoption [10] as well as the perceptions and attitudes of students and practitioners [11-16]. These conversations illustrate that there has been increasing exploration of various facets of AI in radiography, indicating that the emergence of AI technologies will continue to shape the future of radiography and radiography education.

Opinion statements and discussion pieces argue for the inclusion of AI education in undergraduate radiography curricula and appropriate education for current practitioners [3,4,17]. In addition, recommendations were made that higher education institutions must ensure that radiography curricula provide educational opportunities for patient-centered care in relation to AI integration to ensure competent and safe practice [2,3,6]. With increasing automation in clinical tasks, patient-centered care will gain more importance, highlighting the need for academic curricula to prioritize patient-centered care [2,4]. The evolving landscape of radiography, marked by the increasing integration of AI, raises important questions about how to best prepare future radiographers. Therefore, mapping the existing literature on the way that radiography education may need to change because of the integration of AI technologies in clinical practice is needed to grasp the current state of knowledge on the topic for future educational considerations. The aim of this scoping review is to explore and synthesize the literature on the adjustments needed in the radiography curriculum to prepare radiography students for the demands of AI-assisted health care environments.

## Methods

### Overview

The review will follow the Joanna Briggs Institute methodology for scoping reviews because it uses a rigorous and logical approach to scoping reviews [18,19]. The methodological guideline delineates 6 steps to map the extent of the literature on the research topic. These include (1) defining the research question, (2) developing the inclusion and exclusion criteria, (3) describing the search strategy, (4) searching the literature, (5) data extraction, and (6) analyzing the evidence and presenting the results [19]. The scoping review protocol is registered on the Open Science Framework [20]. The PRISMA-ScR (Preferred Reporting Items for Systematic Reviews and Meta-Analyses Extension for Scoping Reviews) reporting guidelines and checklist [21] will be used to report the results (Multimedia Appendix 1).

### Step 1: Defining the Review Question

The research question that will guide the scoping review is “How should the radiography curriculum be adapted to prepare radiography students for the integration of AI technologies in the health care environment?”

For this review, radiography refers to the four different radiography categories, including diagnostic radiography, diagnostic ultrasound, nuclear medicine technology, and radiation therapy.

### Step 2: Study Selection

#### Inclusion Criteria

##### Overview

The types of studies to be considered for this review include quantitative, qualitative, mixed methods studies, scoping, literature, systematic reviews, opinion papers, letters, and conference papers. The selection of study types included in this review is justified based on the need for a comprehensive and nuanced understanding of the topic. The review will consider articles from any country or region, provided that an English translation of the article can be sourced or a translation app is available [22]. The Participants, Concept, and Context (PCC) framework will be used to systematically organize the scope of the review, ensuring it aligns with the objective and the relevant studies are included [18,19].

##### Population

The population consists of radiography students and educators. In addition, articles that form part of multidisciplinary health professions’ groups of which radiography forms a portion will also be included.

##### Concept

The concept focuses on how AI technologies are reshaping the clinical environment and the corresponding adjustments required in the radiography curriculum to prepare radiography students for this evolving landscape.

##### Context

This review will consider articles from academic and clinical settings in all countries.

#### Exclusion Criteria

Studies specifically related to other health professions’ disciplines will be excluded. Furthermore, technologies discussed in the literature that lack AI will be excluded from consideration. All studies that align with the inclusion criteria, including the PCC, will be included in the review. In addition, articles for which the full text is not available through institutional subscriptions, interlibrary loans, or after contacting the corresponding author will not be included in the review. The authors will attempt to maximize access through the available institutional resources, but limitations may still apply. A full list of the eligibility criteria is provided in Table 1.

**Table 1:** Eligibility criteria for the scoping review.

PCC^a^ Framework	Include	Exclude
Population	All 4 radiography categories Radiography educators Radiography students	All other health professions’ disciplines
Concept	Studies that discuss AI^b^ or AI-based tools in the context of radiography	Studies using technology that does not incorporate AI-based tools
Context	Academic and clinical radiography education Undergraduate radiography education Postgraduate radiography education Continuing professional development	Other health professions’ education programs
Study characteristics	The review will consider all types of study designs. Sources printed in all languages Full-text articles	Full-text not available

^a^PCC: Population, Concept, Context.

^b^AI: artificial intelligence.

### Step 3: Search Strategy

The search strategy aimed to include published and unpublished literature. A preliminary search was performed on Scopus to identify articles on the topic in collaboration with a library specialist. The Scopus database was chosen because it is the largest indexing and abstract database and hosts a number of widely acknowledged Radiography journals. The search strategy was developed by examining the text found in the titles and abstracts of the relevant literature and the index terms and keywords used to describe the articles. The process entailed screening the titles and abstracts of 50 articles. Thereafter, the search strategy was refined iteratively through consultations with the librarian and between the authors. The detailed final search strategy for all databases, EBSCOhost, PubMed, Scopus, and Web of Science can be found in Multimedia Appendix 2. Gray literature was retrieved from the databases, including book chapters, conference papers, and letters. The total number of sources found was 2224 (Table 2).

**Table 2:** Electronic databases and gray literature results.

Electronic resources	Sources, n
EBSCO (Academic Search Premier, CINAHL, ERIC)	362
PubMed	1214
Scopus	267
Web of Science	112
Gray literature	269
Results	2224

### Step 4: Selection of Evidence

Following the search, all citations were imported and managed using the Covidence online software [23]. All the duplicates were removed after the studies were imported into the software program. The primary investigator (CK) and a research assistant screened the studies during the title and abstract phase based on the inclusion and exclusion criteria to ensure that they are relevant to the review [24]. The inclusion and exclusion criteria will be piloted on the first 20 articles to ensure that the criteria are applied consistently and that the relevant studies are included.

After the initial screening, a full-text screening of the selected articles will be performed to determine the literature to be included in the review. The reference list of all selected articles will then be hand-searched for additional studies that may be eligible according to the inclusion criteria. Following the full search, all citations will be captured. Any disagreements that arise between the reviewers at each stage of the selection process will be resolved through discussions, or with an additional reviewer and the changes will be noted and shared in the final scoping review report. Furthermore, reasons for the exclusion of sources of evidence at full-text screening will be recorded and reported in the scoping review.

### Step 5: Data Extraction

To meet the aim and research question, the data will be organized according to the Joanna Briggs Institute template source of evidence details, characteristics, and results extraction instrument [22], incorporating key study information such as authors, study population, year of publication, study location, study design, and the key findings (Multimedia Appendix 3). This data extraction instrument was created and will be piloted to minimize potential bias. Piloting of the data extraction instrument will include a selection of 3-5 articles from the scoping review dataset and the two reviewers will perform independent data extraction to ensure that all the necessary data will be captured [21]. The instrument will be used to determine if the inclusion and exclusion criteria have been met during evidence selection. Modifications to the instrument may be made during the course of the literature search and the findings will be detailed in the scoping review report.

### Step 6: Data Analysis

Scoping reviews should include a numerical summary outlining the types of literature that were retrieved and a thematic descriptive analysis [19]. The final themes of the scoping review will be developed using an iterative approach. A narrative description will then be used to synthesize the study findings using descriptive content analysis, and the identified themes will be reported.

## Results

**Figure 1:**
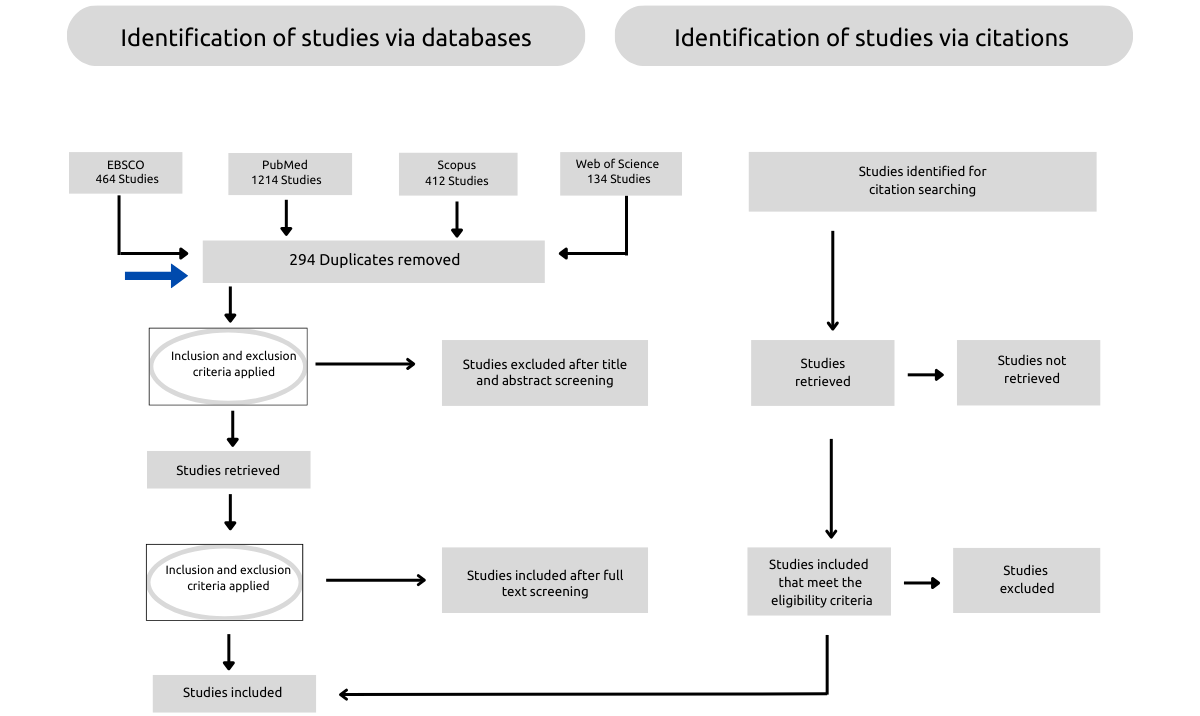
Flowchart of the PRISMA-ScR for the scoping review process. PRISMA-ScR: Preferred Reporting Items for Systematic Reviews and Meta-Analyses Extension for Scoping Reviews.

The final database searches were completed in October 2024 and in total produced 2224 items. Title and abstract screening of 1930 articles is underway after removing the 294 duplicates. The results will be presented in relation to the research question and the objective of the study. Furthermore, the results will be presented in 2 parts as proposed by Peters and colleagues [19]. The first part will consist of the results of the search and the inclusion of the studies will be presented with the PRISMA-ScR flow diagram (Figure 1). The blue arrow indicates the current status. The second part will consist of a narrative description that aligns with the objective of the scoping review. Data analysis and results may be further refined through the review process as the contents of the review are taken into consideration. Finalization of the scoping review is expected by March 2025. The results of the scoping review will be disseminated through publication in an accredited journal.

## Discussion

Addressing the call for an update to the radiography curriculum in response to new technological advancements such as AI is critical to prepare and equip current and future professionals for safe practice. This scoping review will map various studies and is anticipated to provide an overview of the adjustments needed in the radiography curriculum to prepare radiography students for the demands of AI-assisted health care environments. In return, the results will help to inform a variety of stakeholders, including radiography educators, radiography regulatory bodies, and radiography professional groups, to plan for needed changes in radiography education in response to the impact of AI-based technologies on clinical processes. To the best of our knowledge, this is the first scoping review to synthesize existing evidence of how the radiography curriculum might best prepare future practitioners for the demands of AI-assisted health care environments.

A strength of the scoping review is that all available studies of published data will be included. Current available literature focusing on how the radiography curriculum might need to change, considering the incorporation of AI technology in clinical practice, focuses on opinion and discussion pieces, and white papers [4,17,24]. In addition, some studies have highlighted the perceptions of educators and students on the incorporation of AI into radiography curricula; however specific methods of integration and a lack of implementation were noted [1]. Therefore, performing this review is warranted to help provide the necessary insights into changes to the curriculum that are aligned with the advances in technology.

A scoping review explores the breadth and not the depth of a topic, and the reviewers cannot comment on the quality of the studies included in the review. In addition, non-English articles may be excluded from this review, which can potentially lead to missing valuable work. However, for the search strategy, all languages are included and the number of studies for all languages will be documented. This process will show how many studies were identified but not included, promoting transparency in the selection process [22]. Furthermore, where possible translations will be sought to limit the omission of information.

This study will provide valuable insights into how the radiography curriculum should adapt to meet the educational needs of current and future practitioners to ensure competent and safe practice in response to AI technologies. Mapping the existing evidence is essential because the growing integration of AI-based technologies in clinical practice must be supported with appropriate education and training [1,4]. After collating and analyzing the findings of the scoping review, a manuscript containing the final analysis will be written and submitted for publication. In addition, this review will contribute toward a PhD in health professions education.

## Appendix

Multimedia Appendix 1 PRISMA-ScR (Preferred Reporting Items for Systematic Reviews and Meta-Analyses Extension for Scoping Reviews) checklist.

Multimedia Appendix 2 Search strategy.

Multimedia Appendix 3 Data extraction tool.

## Abbreviations

AI: artificial intelligence
PCC: Population, Concept, and Context
PRISMA-ScR: Preferred Reporting Items for Systematic Reviews and Meta-Analyses Extension for Scoping Reviews

## Data Availability

All relevant data are available in the study or as multimedia appendices and will be made available upon request.
