# Supplementary material for: Exploring Curriculum Considerations to Prepare Future Radiographers for an AI-Assisted Health Care Environment: Protocol for Scoping Review
Source: JMIR Res Protoc. 2025 Mar 6;14:e60431. doi: 10.2196/60431 (PMC11926445; doi:10.2196/60431)
Supplement: Multimedia Appendix 2 [file resprot_v14i1e60431_app2.docx]

**Appendix 2: Search strategy**

| **PCC** | **Keywords** | **EBSCO** |
| --- | --- | --- |
| **Population** | (All fields)radiography OR “diagnostic radiography” OR “nuclear medicine” OR “nuclear medicine technology” OR ultrasound OR sonography OR “radiation therapy” OR radiotherapy OR “diagnostic imaging students” OR “diagnostic radiography students” OR “medical radiation science students” OR “medical radiation sciences students” OR “radiologic technology students” OR “radiation therapy students” OR “RT students” OR “radiotherapy students” OR “nuclear medicine students” OR “nuclear medicine technology students” OR “radiologic technology students” OR “sonography students” OR “ultrasound students” OR “diagnostic radiography educators” OR “medical imaging academics” OR “RT educators” OR “radiation therapy educators” OR “radiotherapy educators” OR “nuclear medicine educators” OR “nuclear medicine technology educators” OR “NMT educators” OR “radiologic technology educator” OR “sonography educators” OR “ultrasound educators” OR “clinical instructor” OR “practice educator” OR “clinical tutor” OR “radiography clinical practice educators” OR “clinical educators” OR “clinical placement educators” OR “practice educators” OR “clinical teachers” OR “clinical facilitators” OR academics OR lecturers OR teachers | 3,280,207 |
| **Concept** | (All fields)"artificial intelligence" OR “AI” OR “generative AI” OR “generative artificial intelligence” OR “artificial intelligence technologies” OR "deep learning" OR "machine learning" OR “computer simulation” OR “medical informatics” OR “digital technologies” OR “computer-aided detection” OR “pattern recognition” OR “neural networks” OR “natural language processing” OR “virtual reality” OR “immersive virtual reality” OR “augmented reality” OR “computational intelligence” OR algorithm* OR "image processing computer-assisted" OR "image interpretation computer-assisted" OR "radiographic image interpretation computer-assisted" OR “speech recognition” OR “voice recognition” OR “computer-aided diagnosis” OR “imaging informatics” OR “convolutional neural networks” OR “CNN” OR “artificial neural networks” OR “ANN” OR “AI automation” OR "health care informatics" OR "artificial neural networks" OR ANNs OR "representative learning" OR "digital technologies" OR CAD OR "neural networks" OR "unsupervised machine learning" OR "supervised machine learning" OR "semisupervised learning" OR “semi supervised learning” OR “semi-supervised learning” OR "ML algorithms" OR radiomics | 2,307,287 |
| **Context** | (ALL fields)“medical imaging curriculum” OR “medical imaging education” OR “RT education” OR “radiation therapy education” OR “radiotherapy education” OR “radiation therapy curriculum” OR “RT curriculum” OR “radiography education” OR “radiography curriculum” OR “diagnostic radiography curriculum” OR “diagnostic radiography education” OR “nuclear medicine technology curriculum” OR “nuclear medicine curriculum” OR “nuclear medicine technology education” OR “NMT education” OR “nuclear medicine education” OR “sonography education” OR “ultrasound education” OR “ultrasound curriculum” OR “sonography curriculum” OR “medical radiation sciences education” OR “radiologic technology education” OR “clinical training” OR “practical training” OR “practical skills training” OR “clinical education” | 27,870 |
| **Total** | #1 AND #2 AND #3 | 490 |

| **PCC** | **Keywords** |  |
| --- | --- | --- |
| **Population** | (Title/Abstract)radiography OR “diagnostic radiography” OR “nuclear medicine” OR “nuclear medicine technology” OR ultrasound OR sonography OR “radiation therapy” OR radiotherapy OR “diagnostic imaging students” OR “diagnostic radiography students” OR “medical radiation science students” OR “medical radiation sciences students” OR “radiologic technology students” OR “radiation therapy students” OR “RT students” OR “radiotherapy students” OR “nuclear medicine students” OR “nuclear medicine technology students” OR “radiologic technology students” OR “sonography students” OR “ultrasound students” OR “diagnostic radiography educators” OR “medical imaging academics” OR “RT educators” OR “radiation therapy educators” OR “radiotherapy educators” OR “nuclear medicine educators” OR “nuclear medicine technology educators” OR “NMT educators” OR “radiologic technology educator” OR “sonography educators” OR “ultrasound educators” OR “clinical instructor” OR “practice educator” OR “clinical tutor” OR “radiography clinical practice educators” OR “clinical educators” OR “clinical placement educators” OR “practice educators” OR “clinical teachers” OR “clinical facilitators” OR academics OR lecturers OR teachers  "radiography"[MeSH Terms] OR "radionuclide imaging"[MeSH Terms] OR "nuclear medicine"[MeSH Terms] OR "ultrasonography"[MeSH Terms] OR "radiotherapy"[MeSH Terms] | 2,401,234 |
| **Concept** | (Title/Abstract)"artificial intelligence" OR “AI” OR “generative AI” OR “generative artificial intelligence” OR “artificial intelligence technologies” OR "deep learning" OR "machine learning" OR “computer simulation” OR “medical informatics” OR “digital technologies” OR “computer-aided detection” OR “pattern recognition” OR “neural networks” OR “natural language processing” OR “virtual reality” OR “immersive virtual reality” OR “augmented reality” OR “computational intelligence” OR algorithm* OR "image processing computer-assisted" OR "image interpretation computer-assisted" OR "radiographic image interpretation computer-assisted" OR “speech recognition” OR “voice recognition” OR “computer-aided diagnosis” OR “imaging informatics” OR “convolutional neural networks” OR “CNN” OR “artificial neural networks” OR “ANN” OR “AI automation” OR "health care informatics" OR "artificial neural networks" OR ANNs OR "representative learning" OR "digital technologies" OR CAD OR "neural networks" OR "unsupervised machine learning" OR "supervised machine learning" OR "semisupervised learning" OR “semi supervised learning” OR “semi-supervised learning” OR "ML algorithms" OR radiomics  Artificial Intelligence [MeSH Terms] | 853,447 |
| **Context** | (Title/Abstract)“medical imaging curriculum” OR “medical imaging education” OR “RT education” OR “radiation therapy education” OR “radiotherapy education” OR “radiation therapy curriculum” OR “RT curriculum” OR “radiography education” OR “radiography curriculum” OR “diagnostic radiography curriculum” OR “diagnostic radiography education” OR “nuclear medicine technology curriculum” OR “nuclear medicine curriculum” OR “nuclear medicine technology education” OR “NMT education” OR “nuclear medicine education” OR “sonography education” OR “ultrasound education” OR “ultrasound curriculum” OR “sonography curriculum” OR “medical radiation sciences education” OR “radiologic technology education” OR “clinical training” OR “practical training” OR “practical skills training” OR “clinical education”  Curriculum [MeSH Terms] OR Education [MeSH Terms] OR Preceptorship [MeSH Terms] | 984,386 |
| **Total** | #1 AND #2 AND #3 | 1214 |

| **PCC** | **Keywords** | **Scopus** |
| --- | --- | --- |
| **Population** | (Title, abstract, keywords)radiography OR “diagnostic radiography” OR “nuclear medicine” OR “nuclear medicine technology” OR ultrasound OR sonography OR “radiation therapy” OR radiotherapy OR “diagnostic imaging students” OR “diagnostic radiography students” OR “medical radiation science students” OR “medical radiation sciences students” OR “radiologic technology students” OR “radiation therapy students” OR “RT students” OR “radiotherapy students” OR “nuclear medicine students” OR “nuclear medicine technology students” OR “radiologic technology students” OR “sonography students” OR “ultrasound students” OR “diagnostic radiography educators” OR “medical imaging academics” OR “RT educators” OR “radiation therapy educators” OR “radiotherapy educators” OR “nuclear medicine educators” OR “nuclear medicine technology educators” OR “NMT educators” OR “radiologic technology educator” OR “sonography educators” OR “ultrasound educators” OR “clinical instructor” OR “practice educator” OR “clinical tutor” OR “radiography clinical practice educators” OR “clinical educators” OR “clinical placement educators” OR “practice educators” OR “clinical teachers” OR “clinical facilitators” OR academics OR lecturers OR teachers | 3,573,058 |
| **Concept** | (Title, abstract, keywords)"artificial intelligence" OR “AI” OR “generative AI” OR “generative artificial intelligence” OR “artificial intelligence technologies” OR "deep learning" OR "machine learning" OR “computer simulation” OR “medical informatics” OR “digital technologies” OR “computer-aided detection” OR “pattern recognition” OR “neural networks” OR “natural language processing” OR “virtual reality” OR “immersive virtual reality” OR “augmented reality” OR “computational intelligence” OR algorithm* OR "image processing computer-assisted" OR "image interpretation computer-assisted" OR "radiographic image interpretation computer-assisted" OR “speech recognition” OR “voice recognition” OR “computer-aided diagnosis” OR “imaging informatics” OR “convolutional neural networks” OR “CNN” OR “artificial neural networks” OR “ANN” OR “AI automation” OR "health care informatics" OR "artificial neural networks" OR ANNs OR "representative learning" OR "digital technologies" OR CAD OR "neural networks" OR "unsupervised machine learning" OR "supervised machine learning" OR "semisupervised learning" OR “semi supervised learning” OR “semi-supervised learning” OR "ML algorithms" OR radiomics | 7,599,492 |
| **Context** | (Title, abstract, keywords)“medical imaging curriculum” OR “medical imaging education” OR “RT education” OR “radiation therapy education” OR “radiotherapy education” OR “radiation therapy curriculum” OR “RT curriculum” OR “radiography education” OR “radiography curriculum” OR “diagnostic radiography curriculum” OR “diagnostic radiography education” OR “nuclear medicine technology curriculum” OR “nuclear medicine curriculum” OR “nuclear medicine technology education” OR “NMT education” OR “nuclear medicine education” OR “sonography education” OR “ultrasound education” OR “ultrasound curriculum” OR “sonography curriculum” OR “medical radiation sciences education” OR “radiologic technology education” OR “clinical training” OR “practical training” OR “practical skills training” OR “clinical education” | 33,341 |
| **Total** |  | 412 |

| **PCC** | **Keywords** | **Web of Science** |
| --- | --- | --- |
| **Population** | (Title, abstract, keywords)radiography OR “diagnostic radiography” OR “nuclear medicine” OR “nuclear medicine technology” OR ultrasound OR sonography OR “radiation therapy” OR radiotherapy OR “diagnostic imaging students” OR “diagnostic radiography students” OR “medical radiation science students” OR “medical radiation sciences students” OR “radiologic technology students” OR “radiation therapy students” OR “RT students” OR “radiotherapy students” OR “nuclear medicine students” OR “nuclear medicine technology students” OR “radiologic technology students” OR “sonography students” OR “ultrasound students” OR “diagnostic radiography educators” OR “medical imaging academics” OR “RT educators” OR “radiation therapy educators” OR “radiotherapy educators” OR “nuclear medicine educators” OR “nuclear medicine technology educators” OR “NMT educators” OR “radiologic technology educator” OR “sonography educators” OR “ultrasound educators” OR “clinical instructor” OR “practice educator” OR “clinical tutor” OR “radiography clinical practice educators” OR “clinical educators” OR “clinical placement educators” OR “practice educators” OR “clinical teachers” OR “clinical facilitators” OR academics OR lecturers OR teachers | 1,757,944 |
| **Concept** | (Title, abstract, keywords)"artificial intelligence" OR “AI” OR “generative AI” OR “generative artificial intelligence” OR “artificial intelligence technologies” OR "deep learning" OR "machine learning" OR “computer simulation” OR “medical informatics” OR “digital technologies” OR “computer-aided detection” OR “pattern recognition” OR “neural networks” OR “natural language processing” OR “virtual reality” OR “immersive virtual reality” OR “augmented reality” OR “computational intelligence” OR algorithm* OR "image processing computer-assisted" OR "image interpretation computer-assisted" OR "radiographic image interpretation computer-assisted" OR “speech recognition” OR “voice recognition” OR “computer-aided diagnosis” OR “imaging informatics” OR “convolutional neural networks” OR “CNN” OR “artificial neural networks” OR “ANN” OR “AI automation” OR "health care informatics" OR "artificial neural networks" OR ANNs OR "representative learning" OR "digital technologies" OR CAD OR "neural networks" OR "unsupervised machine learning" OR "supervised machine learning" OR "semisupervised learning" OR “semi supervised learning” OR “semi-supervised learning” OR "ML algorithms" OR radiomics | 2,787,643 |
| **Context** | (Title, abstract, keywords)“medical imaging curriculum” OR “medical imaging education” OR “RT education” OR “radiation therapy education” OR “radiotherapy education” OR “radiation therapy curriculum” OR “RT curriculum” OR “radiography education” OR “radiography curriculum” OR “diagnostic radiography curriculum” OR “diagnostic radiography education” OR “nuclear medicine technology curriculum” OR “nuclear medicine curriculum” OR “nuclear medicine technology education” OR “NMT education” OR “nuclear medicine education” OR “sonography education” OR “ultrasound education” OR “ultrasound curriculum” OR “sonography curriculum” OR “medical radiation sciences education” OR “radiologic technology education” OR “clinical training” OR “practical training” OR “practical skills training” OR “clinical education” | 11,906 |
| **TOTAL** | #1 AND #2 AND #3 | 133 |
