# Supplementary material for: Exploring Curriculum Considerations to Prepare Future Radiographers for an AI-Assisted Health Care Environment: Protocol for Scoping Review
Source: JMIR Res Protoc. 2025 Mar 6;14:e60431. doi: 10.2196/60431 (PMC11926445; doi:10.2196/60431)
Supplement: Multimedia Appendix 3 [file resprot_v14i1e60431_app3.docx]

**Appendix 3: Draft data extraction tool**

| Citation details | Year of publication | Study objective | Study location | Research design | Population and sample size | Key findings related to the influence of AI on radiography education | Influence of AI on the radiographer’s role | Tension between efficiency and patient-centred care | Educational needs for current and future radiographers | Influence of AI on patient-centred care education |
| --- | --- | --- | --- | --- | --- | --- | --- | --- | --- | --- |
